# Short-term outcome and prognostic factors in acute fulminant myocarditis with acute kidney injury patients

**DOI:** 10.3389/fmed.2026.1713936

**Published:** 2026-03-03

**Authors:** Mengyuan Zhu, Zhenzhen You, Lijiao Wang, Yue Gu, Xiaoguang Fan

**Affiliations:** 1 Department of Nephrology, Fuwai Central China Cardiovascular Hospital, Central China Fuwai Hospital of Zhengzhou University, Zhengzhou, Henan, China; 2 Department of Nephrology, Henan Provincial People’s Hospital, Henan Key Laboratory for Nephrology, Zhengzhou, Henan, China

**Keywords:** acute fulminant myocarditis, AKI, prognostic factors, short-term outcome, SIRI

## Abstract

**Background:**

To evaluate the impact of the systemic inflammatory response index (SIRI) and other clinical features on short-term outcome in patients with acute fulminant myocarditis (AFM) complicated by acute kidney injury (AKI).

**Methods:**

We retrospectively analyzed patients diagnosed with AFM and AKI at Fuwai Central China Cardiovascular Hospital between March 2018 and September 2024. Patients were divided into survival and death groups according to 30-day mortality. Demographic data, vital signs, laboratory parameters, echocardiographic findings, and treatment details were compared between the two groups. Multivariate logistic regression analysis was performed to examine independent predictors of 30-day mortality, and receiver operating characteristic (ROC) curve analysis was performed to assess their predictive value.

**Results:**

A total of 118 patients were included (median age: 41 years; 52.5% male). The death group (*n* = 44) had higher rates of respiratory failure and use of extracorporeal membrane oxygenation, mechanical ventilation and continuous renal replacement therapy than the survival group (*n* = 74). Proteinuria, leukocyte and neutrophil counts, neutrophil-lymphocyte ratio (NLR), SIRI, troponin, creatine kinase, lactate dehydrogenase, aspartate aminotransferase, and serum creatinine were significantly higher in the death group, while 24-h urine volume, ejection fraction (EF), blood pressure, and hospitalization time were lower (all *p* < 0.05). Leukocyte count, neutrophil count, NLR, and SIRI correlated positively with 30-day mortality. Multivariate analysis identified 24-h urine volume (OR 0.999, 95%CI 0.999–1.000, *p* = 0.036) and EF (OR 0.944, 95%CI 0.896–0.995, *p* = 0.032) as independent predictors. ROC curve analysis showed good predictive value (AUC 0.819 for urine volume and 0.740 for EF).

**Conclusion:**

SIRI was associated with 30-day mortality in AFM and AKI, while only reduced 24-h urine volume and EF were independent prognostic markers.

## Introduction

Acute fulminant myocarditis (AFM) is a sudden-onset and rapidly progressive myocardial disease. It can be secondary to or accompanied by respiratory failure and acute kidney injury (AKI), conditions which often necessitate mechanical circulatory support (MCS). According to the China expert consensus, comprehensive management of AFM should be based on life support measures such as extracorporeal membrane oxygenation (ECMO), intra-aortic balloon pump (IABP), continuous renal replacement therapy (CRRT), pacemaker implantation, and mechanical ventilation, in combination with immunotherapy using adequate doses of glucocorticoids and immunoglobulin to modulate the immune response. Advances in MCS technology and standardization of treatment have helped reduce the in-hospital mortality rate of AFM to 3.7–8.1% (1, 2). However, AFM complicated by AKI remains associated with poor outcomes. A recent study reported a 30-day mortality rate of 40.7% in AFM patients with renal complications, significantly higher than that of the overall AFM population (3). Therefore, it is imperative to identify reliable biomarkers that can help predict short-term outcomes in patients with AFM and AKI.

The pathogenesis of AFM involves excessive activation of the heart’s innate immunity and the development of an inflammatory storm (2). Viral infections or autoimmune disease can trigger excessive immune activation and cytokine release, leading to severe cardiomyocyte injury, cardiac remodeling, and ultimately AFM and death. In recent years, multiple inflammatory factors have been implicated in this process. The systemic inflammatory response index (SIRI), defined as neutrophil × monocyte/lymphocyte count, has emerged as a novel composite marker of inflammation. Previous studies have demonstrated its association with AKI and related mortality (4–6). For example, SIRI has been shown to predict the incidence of AKI and in-hospital mortality in critically ill pediatric patients and to serve as an independent risk factor for AKI and death in patients with abdominal trauma. Moreover, a retrospective study reported that SIRI was a reliable predictor of 30-day and 1-year mortality in patients with sepsis-related AKI, supporting its value as a comprehensive prognostic biomarker.

However, few studies have examined the relationship between SIRI and short-term prognosis in AFM and AKI patients. Therefore, we investigated the impact of SIRI and other clinical features on 30-day mortality in this population, with the goal of developing a prognostic model for short-term outcomes.

## Materials and methods

### Study design and participants

This was a retrospective study. AFM and AKI are defined according to the Chinese Expert Consensus (1, 7).

(1) The diagnostic criteria for AFM: a sudden onset with prodromal symptoms such as fever, fatigue, poor appetite, or diarrhea, or chest tightness and pain, followed by rapid development of severe hemodynamic disturbances (including hypotension or shock) or serious arrhythmias (malignant arrhythmias like atrioventricular block, sinus tachycardia, ventricular tachycardia or ventricular fibrillation); significantly elevated myocardial injury markers such as hscTnI/cTnI and BNP/NT-proBNP, along with marked ECG changes (low voltage, widespread ST-segment and T-wave alterations and conduction blocks); echocardiography reveals diffuse reduced ventricular wall motion, significantly decreased left ventricular ejection fraction and decreased left ventricular long-axis strain; elevated inflammatory levels, particularly sST2 and CMRI imaging indicating myocardial edema, congestion, capillary exudation, necrosis or fibrosis. When excluding acute myocardial infarction and stress cardiomyopathy, a clinical diagnosis of AFM can be confirmed.
(2) AKI is defined as any of the following: Increase in SCr by ≥0.3 mg/dL (≥26.5 mmol/L) within 48 h; or Increase in SCr to ≥1.5 times baseline, which is known or presumed to have occurred within the prior 7 days; or Urine volume <0.5 mL/kg/h for 6 h. AKI 1–3 stage were all included in this study.

(3) Initiate CRRT emergently when life-threatening changes including severe hyperkalemia, severe acidosis, pulmonary edema, and uremic complications. When the patient was diagnosed as AKI stage 2 or stage 3, CRRT should also be considered.

Patients diagnosed with both AFM and AKI at the Fuwai Central China Cardiovascular Hospital (Cardiac Center of Henan Provincial People’s Hospital) between March 2018 and September 2024 were eligible for inclusion. Patients were divided into a survival group and a death group based on 30-day mortality.

Exclusion criteria were as follows: (1) pre-existing chronic kidney failure before admission, and (2) incomplete baseline data. The study was approved by the Hospital Ethics Committee of Henan Provincial People’s Hospital (Approval No: 2019 Lunshen No. 06).

### Data collection

For each patient, demographic characteristics, vital signs, laboratory parameters, echocardiographic findings, and treatment regimens were obtained. Vital signs comprised 24-h urine volume after admission, blood pressure, and heart rate (HR) at admission. Laboratory parameters included routine tests (hematology, biochemistry, and liver function), cardiac biomarkers such as myoglobin (MYO), troponin, B-type natriuretic peptide (BNP), creatine kinase (CK), lactate dehydrogenase (LDH), aspartate aminotransferase (AST), as well as renal function indicators, including proteinuria, serum creatinine (Scr) on admission, and peak Scr. Inflammatory markers, including NLR, SIRI, C-reactive protein (CRP), and procalcitonin (PCT), were also recorded. Echocardiographic measurements included left ventricular end-diastolic volume (LVEDV), left ventricular end-diastolic dimension (LVDD), interventricular septal thickness (ISV), and ejection fraction (EF). Treatment data were collected on the use of glucocorticoids, intravenous immunoglobulin (IVIG), ECMO, IABP, CRRT, pacemaker implantation, and mechanical ventilation.

### Statistical analysis

The data were analyzed using SPSS 21. Continuous variables with a normal distribution are presented as mean ± standard deviation (SD), and compared between groups using the independent samples *t*-test. Continuous variables with a non-normal distribution are expressed as median and interquartile range [M (Q1, Q3)] and compared using the Mann–Whitney *U* test. Categorical variables are expressed as frequency (%), and between-group differences were assessed using the chi-squared test or Fisher’s exact test, as appropriate. Multivariate logistic regression analysis was conducted to identify independent predictors of 30-day mortality. The predictive value of relevant factors was assessed by receiver operating characteristic (ROC) curve analysis. The Area Under the Curve (AUC) were compared using DeLong’s test. Spearman correlation analysis was applied to test the correlation between inflammatory indicators and 30-day mortality. *p* < 0.05 was considered indicative of statistical significance.

## Results

### Clinical characteristics of the study population

**Figure 1:**
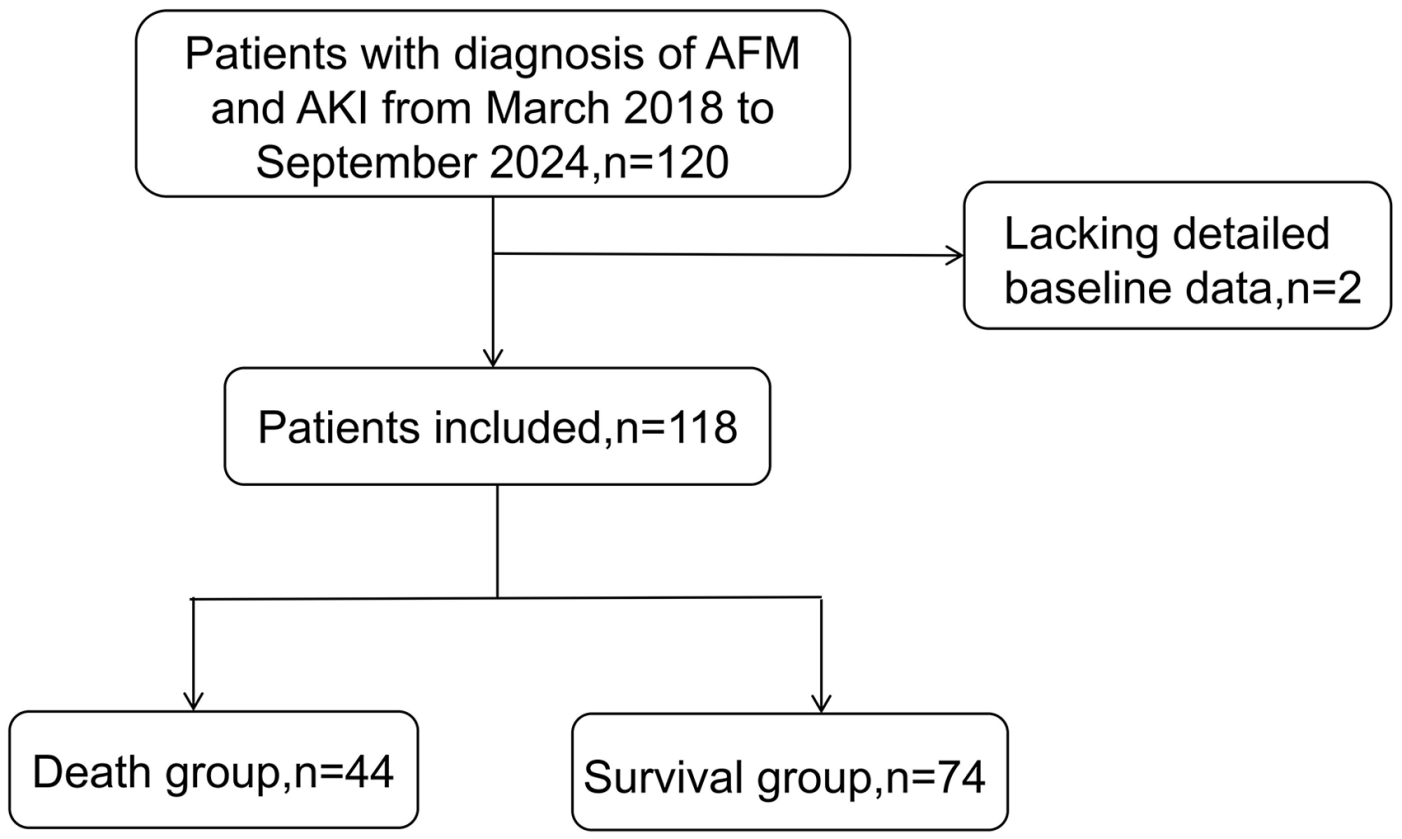
Flow chart of patient selection.

Of the 120 patients initially screened, 2 were excluded due to incomplete baseline data. Finally, 118 patients with AFM and AKI were included in the analysis. The median age was 41 years, and 62 patients (52.5%) were male. Based on 30-day outcomes, patients were divided into the death group (*n* = 44, 37.3%) and the survival group (*n* = 74, 62.7%). The enrollment process is depicted in Figure 1, and the baseline characteristics are summarized in Table 1.

**Table 1:** Comparison of clinical characteristics of patients in the survival and death groups.

Variable	Survival group (*n* = 74)	Death group (*n* = 44)	*t*/*Z*/*χ*^2^	*p* value
Demographics				
Male, *n* (%)	41 (55.4)	21 (47.7)	0.652	0.419
Age, years	47.5 (27.5, 59)	40 (23.5, 51.8)	−0.844	0.399
BMI (kg/m^2^)	23.3 (19.7, 25.4)	23 (20.2, 24.8)	−0.061	0.951
Hospitalization time, days	17 (12, 27.3)	5 (2, 11.5)	−6.402	<0.001
Comorbidities				
Diabetes, *n* (%)	8 (10.8)	3 (6.8)	0.155	0.694
CAD, *n* (%)	17 (23)	5 (11.4)	2.452	0.117
Respiratory failure, *n* (%)	19 (25.7)	20 (45.5)	4.878	0.027
Vital signs at admission				
SBP, mmHg	102.5 (91.8, 117.3)	95 (78.5, 111.3)	−2.369	0.018
DBP, mmHg	66 (58.5, 74.3)	58.5 (44.3, 76.5)	−2.216	0.027
HR, beats/min	99 (81.5, 122.3)	110 (85.5, 133)	−0.968	0.333
24-h urine volume, mL	2,760 (1,750, 3,832.5)	315 (22.5, 2,000)	−5.78	<0.001
Inflammatory markers				
Leukocyte count, 10^9^/L	12.2 (8.8, 16.1)	15.2 (10, 19.9)	−1.981	0.048
Neutrophil count, 10^9^/L	9.9 (6.9, 13.5)	13.1 (8.7, 17.8)	−2.273	0.023
NLR	9.1 (5.3, 14.2)	14.1 (9, 22.1)	−2.766	0.006
SIRI	4 (2.4, 8)	8 (3.1, 14.1)	−2.429	0.015
CRP, mg/L	48 (11.1, 99.1)	30.9 (15.2, 71.3)	−0.991	0.322
PCT, ng/mL	1.4 (0.3, 8.8)	2.7 (0.5, 8.2)	−1.241	0.214
Renal markers				
Proteinuria, plus	0 (0, 1)	1 (0, 2)	−2.965	0.003
BUN, mmol/L	8.8 (5.7, 12.6)	9.1 (6.8, 14.2)	−1.177	0.239
UA, μmol/L	439.5 (294.5, 624.3)	564.5 (421.8, 783.8)	−2.863	0.004
Scr on admission, μmol/L	107 (71, 137.3)	147 (93.5, 207.3)	−2.958	0.003
Peak Scr, μmol/L	137 (101.3, 224.5)	205.5 (135.3, 298)	−2.663	0.008
AKI stage			50.384	<0.001
Stage 1, *n* (%)	43 (58.1)	6 (13.6)		
Stage 2, *n* (%)	23 (31.1)	5 (11.4)		
Stage 3, *n* (%)	8 (10.8)	33 (75)		
Cardiac biomarkers				
AST, U/L	270.5 (68, 891)	694 (226.3, 2,697.8)	−2.677	0.007
CK, U/L	519 (190.3, 1,526)	1,563 (634, 5,386.5)	−3.114	0.002
LDH, U/L	723.5 (378.5, 1,703)	1,617 (556.3, 4,515.5)	−3.008	0.003
TNI, ng/mL	8.2 (0.8, 21.1)	13.6 (2.9, 30)	−1.996	0.046
BNP, pg./mL	1,450 (801.3, 2,237.5)	1,059 (168.3, 2,715)	−0.921	0.357
Echocardiography				
LVDD, mm	48 (43.8, 54)	47 (40, 54)	−0.883	0.377
LVEDV, mL	109 (86.5, 141)	98 (71.3, 141.8)	−1.269	0.204
IVST, mm	9 (8, 10)	9 (8, 11)	−0.532	0.595
EF, %	29.5 (20, 40)	20 (16, 25)	−4.355	<0.001
Medications				
Glucocorticoids, *n* (%)	60 (81.1)	36 (81.8)	0.01	0.921
IVIG, *n* (%)	66 (89.2)	38 (86.4)	0.211	0.646
Life support treatment				
ECMO, *n* (%)	42 (56.8)	39 (88.6)	13.029	<0.001
IABP, *n* (%)	44 (59.5)	29 (65.9)	0.487	0.485
Pacemaker, *n* (%)	12 (16.2)	5 (11.4)	0.379	0.538
Ventilator, *n* (%)	34 (45.9)	42 (95.5)	27.383	<0.001
CRRT, *n* (%)	21 (28.4)	33 (75)	24.164	<0.001

BMI, body mass index; CAD, coronary artery disease; SBP, systolic blood pressure; DBP, diastolic blood pressure; HR, heart rate; NLR, neutrophil-lymphocyte ratio; SIRI, systemic inflammatory response index; CRP, C-reactive protein; PCT, procalcitonin; BUN, urea nitrogen; UA, uric acid; Scr, serum creatinine; AKI, acute kidney injury; AST, aspartate transaminase; CK, creatine kinase; LDH, lactate dehydrogenase; TNI, troponin; BNP, B-type natriuretic peptide; LVDD, left ventricular end-diastolic diameter; LVEDV, left ventricular end-diastolic volume; IVST, interventricular septal thickness, EF, ejection fraction; IVIG, intravenous immunoglobulin; ECMO, extracorporeal membrane oxygenation; IABP, intra-aortic balloon pump; CRRT, continuous renal replacement therapy.

No significant differences were observed in sex, age, or body mass index (BMI) between the two groups. The death group had a higher incidence of respiratory failure and worse AKI stage. Due to disease severity, this group also had greater use of ECMO, mechanical ventilation and CRRT. Laboratory analyses showed that levels of proteinuria, leukocytes, neutrophils, NLR, SIRI, troponin, CK, LDH, AST, Scr on admission, and peak Scr were all significantly higher in the death group (all *p* < 0.05). Hospitalization time, total carbon dioxide, SBP, DBP, 24-h urine volume after admission, and EF were significantly lower in the death group (all *p* < 0.05).

### Correlation between inflammatory biomarkers and 30-day mortality

The levels of inflammatory biomarkers, including leukocytes, neutrophils, NLR, and SIRI, were all positively correlated with 30-day mortality in patients with AFM and AKI (Table 2).

**Table 2:** Correlation between inflammatory biomarkers and 30-day mortality in patients with AFM and AKI.

Variables	Spearman correlation	*p* value
Leukocyte-mortality	0.183	0.047
Neutrophil-mortality	0.210	0.022
NLR-mortality	0.256	0.005
SIRI-mortality	0.225	0.014

NLR, neutrophil-lymphocyte ratio; SIRI, systemic inflammatory response index.

### Risk factors for 30-day mortality in AFM with AKI

**Figure 2:**
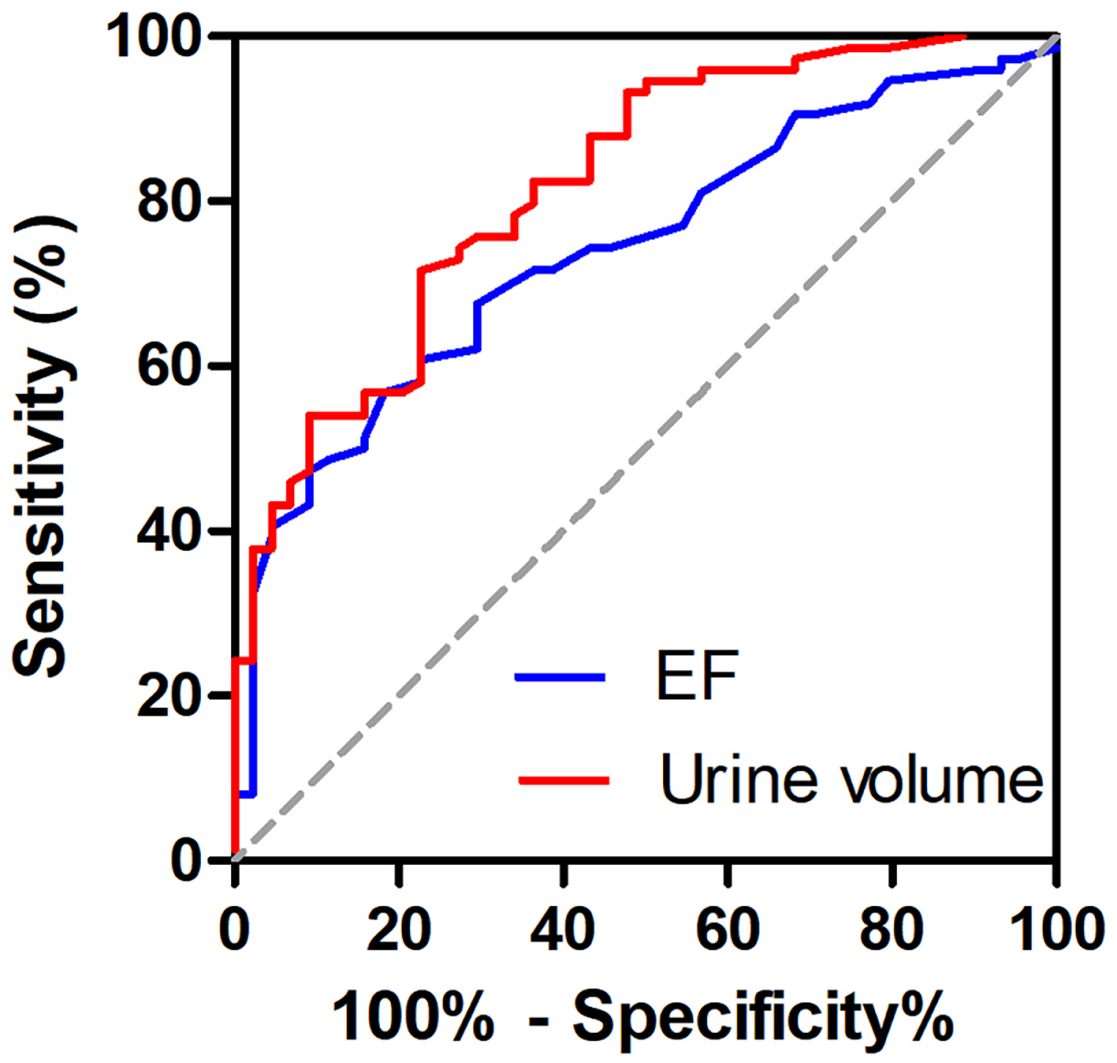
Receiver operating characteristic (ROC) curves of 24-h urine volume after admission and ejection fraction (EF) for predicting 30-day mortality in patients with AFM and AKI.

Univariate analysis identified several potential risk factors for 30-day mortality, including SBP, 24-h urine volume after admission, proteinuria, leukocyte count, neutrophil count, NLR, SIRI, Scr on admission, LDH, AST, EF, and the presence of respiratory failure. After the assessment of collinearity, neutrophils count and AST were excluded. Multivariate logistic regression analysis identified 24-h urine volume (OR 0.999, 95%CI 0.999–1.000, *p* = 0.036) and EF (OR 0.944, 95%CI 0.896–0.995, *p* = 0.032) as independent risk factors for 30-day mortality (Table 3). ROC curve analysis showed that the area under the curve (AUC) for 24-h urine volume after admission was 0.819 (95% CI 0.743–0.895) and for EF was 0.740 (95% CI 0.651–0.829), and there was no significant difference between the AUCs of 24-h urine volume and EF (*p* = 0.17). The optimal cut-off values were 2025 mL for 24-h urine volume and 27.5% for EF (Figure 2 and Table 4).

**Table 3:** Logistic regression analysis of predictors for 30-day mortality in patients with AFM and AKI.

Variables	Univariable analysis	*p* value	Multivariable analysis	*p* value
	Unadjusted OR (95% CI)		Adjusted OR (95% CI)	
SBP	0.976 (0.957, 0.995)	0.013		
24-h urine volume	0.999 (0.999, 0.999)	<0.001	0.999 (0.999, 1.000)	0.036
Proteinuria	1.965 (1.200, 2.396)	0.003		
Leukocyte	1.078 (1.011, 1.149)	0.021		
Neutrophil	1.086 (1.013, 1.163)	0.02		
NLR	1.046 (1.004, 1.089)	0.032		
SIRI	1.084 (1.024, 1.148)	0.006		
Scr on admission	1.005 (1.001, 1.009)	0.028		
LDH	1.000 (1.000, 1.000)	0.008		
AST	1.000 (1.000, 1.000)	0.016		
EF	0.917 (0.876, 0.959)	<0.001	0.944 (0.896, 0.995)	0.032
Respiratory failure	0.288 (0.131, 0.634)	0.002		
ECMO	5.943 (2.104, 16.787)	0.001		
Ventilator	24.706 (5.566, 109.663)	<0.001		
CRRT	7.571 (3.239, 17.699)	<0.001		

SBP, systolic blood pressure; NLR, neutrophil-lymphocyte ratio; SIRI, systemic inflammatory response index; Scr, serum creatinine; LDH, lactate dehydrogenase; AST, aspartate transaminase; EF, ejection fraction; ECMO, extracorporeal membrane oxygenation; CRRT, continuous renal replacement therapy.

**Table 4:** AUC for predictors of 30-day mortality in patients with AFM and AKI.

Variables	AUC (95% CI)	Cut-off value	Sensitivity (%)	Specificity (%)
24-h urine volume	0.819 (0.743, 0.895)	2,025	0.716	0.773
EF	0.740 (0.651, 0.829)	27.5	0.568	0.818

EF, ejection fraction.

## Discussion

AFM leads to multiple organ failure and often needs MCS. Numerous studies have demonstrated that concomitant AKI worsens prognosis in AFM. Hao et al. found that AFM patients with renal complications had a 30-day mortality rate of 40.7%, more than threefold higher than those without renal complications (3). Similarly, Ho et al. observed that within 24 h of ECMO support, patients in the death group experienced a 108% increase in Scr, whereas survivors showed an 8.5% decrease. An increase in Scr within 24 h of ECMO support was associated with an increased in-hospital mortality in AFM patients (8). Other studies have shown that higher rates of complications, elevated baseline Scr and estimated glomerular filtration rate (eGFR), the most severe stage of eGFR, and abnormal echocardiographic findings are predictors of increased mortality in patients with acute myocarditis. Notably, eGFR and left atrial diameter were identified as independent predictors of in-hospital mortality in these patients (9).

In our cohort, the 30-day mortality rate of AFM with AKI was 37%. Patients in the death group had higher rates of respiratory failure as well as greater use of ECMO, mechanical ventilation and CRRT, reflecting their more critical condition. Furthermore, levels of proteinuria, Scr at admission, and peak Scr were significantly higher, while total carbon dioxide, 24-h urine output after admission were lower, suggesting poorer baseline renal function, more severe renal injury, metabolic acidosis, and more pronounced oliguria in the death group. In addition, the death group exhibited higher levels of troponin, CK, LDH, and AST, alongside lower SBP, DBP, and EF. These findings indicate more severe myocardial injury, impaired cardiac function, and circulatory instability in non-survivors. Collectively, our results highlight the importance of early recognition and correction of renal dysfunction and myocardial injury to improve outcomes in AFM with AKI.

Innate immune hyperactivation triggered by various etiologies, together with the cytokine storm caused by the rapid release of inflammatory mediators from immune cells, is considered a major driver of the acute onset, rapid progression, severe clinical course, and high mortality of fulminant myocarditis (10, 11). The formation and mechanisms of the cytokine storm are complex. Fundamentally, cardiac injury arises from the imbalance between pro-inflammatory response and anti-inflammatory responses, leading to excessive immune activation and the development of an inflammatory storm (12, 13). This dysregulated immune response creates a positive feedback loop between immune and non-immune cells and cytokines, resulting in excessive cytokine release.

Experimental studies have further elucidated the mechanisms. In AFM myocardial biopsy specimens, innate immune cells (primarily macrophages and neutrophils) predominate during the acute phase (14). Remels et al. found that cytokines secreted from infiltrating leukocytes activate NF-κB signalling in cardiomyocytes thereby modulating myocardial energy metabolism in acute viral myocarditis (15). In an AFM mouse model, peripheral blood neutrophils were shown to migrate to the myocardium via the Cxcl2/Cxcl3 pathway, leading to acute neutrophil accumulation in the heart and phenotypic alterations in cardiomyocytes. Neutrophils also amplified the cytokine storm by recruiting and activating pro-inflammatory monocytes (16). Inhibiting extracellular trap of neutrophil formation could reduce inflammation, maintained systolic function and improved the pathological phenotype in the acute phase of viral myocarditis mice (17, 18). Other investigators have reported that NLR and monocyte-to-lymphocyte ratio (MLR) predict hospital stay duration in myocarditis; NLR was comparable to established high-risk models in predicting mortality and heart transplantation in acute myocarditis (19, 20). As a novel composite marker of systemic inflammation, SIRI has been shown to predict prognosis in critically ill patients with AKI in several studies (3–5). Moreover, it was associated with the severity of acute myocarditis in adults and could independently predict adverse myocarditis prognoses in children (21, 22).

In our study, leukocyte count, neutrophil count, NLR, and SIRI were all higher in the death group compared with the survival group, and these inflammatory biomarkers were positively correlated with 30-day mortality. These findings are consistent with the cytokine storm hypothesis in AFM and support the role of SIRI as a marker of inflammatory injury in patients with AFM and AKI.

Early identification of adverse prognosis in AFM complicated by AKI is particularly important and should be based on comprehensive clinical indicators. Several studies have examined mortality predictors in AFM. In pediatric patients with severe heart disease (including AFM) receiving ECMO treatment, factors such as male sex, bleeding, kidney injury, and central catheterization were associated with an increased short-term mortality (23). In children with AFM, MYO levels and EF have been identified as prognostic markers that reflect disease severity, with greater diagnostic and predictive value when combined (24). Among AFM patients receiving ECMO, elevated 24-h serum lactate and peak serum troponin I levels have been linked to in-hospital mortality (25). Thyroid function has also been implicated: low triiodothyronine syndrome and free triiodothyronine independently predicted 30-day mortality in AFM patients (26). However, few studies have specifically addressed the risk factors for 30-day mortality in AFM patients complicated by AKI, therefore the predictive model in our study incorporated basic vital signs, cardiac injury indicators, renal injury indicators, and related inflammatory factors based on existing research findings.

After the assessment of collinearity, multivariable logistic regression analysis identified 24-h urine volume after admission and EF as independent risk factors for 30-day mortality. ROC curve analysis demonstrated that a cutoff of 2025 mL for 24-h urine volume after admission yielded an AUC of 0.819 (95% CI 0.743–0.895, *p* < 0.05), with 71.6% sensitivity and 77.3% specificity. For EF, a cutoff of 27.5% yielded an AUC of 0.740 (95% CI 0.651–0.829, *p* < 0.05), with 56.8% sensitivity and 81.8% specificity. What’s more, there was no statistically difference between the AUCs of 24-h urine volume after admission and EF (*p* = 0.17), indicating that they had the similar predictive value for the short-term mortality in AFM with AKI, which was consistent with previous reports (27). We speculate that optimizing cardiac function, renal function, and inflammatory status may improve the poor prognosis of patients with AFM complicated by AKI.

Our study still has some limitations. Firstly, it was retrospective in design and included a small sample size. Secondly, the absence of myocardial biopsy limited pathological confirmation. Thirdly, although SIRI was associated with 30-day mortality in AFM patients with AKI, it does not remain an independent predictor, the role of SIRI require further investigation. Finally, this study focused on 30-day mortality while lacking long-term prognosis assessment. Therefore, our findings should be verified in larger cohorts with long-term follow-up and mechanistic studies.

## Conclusion

Our findings demonstrated SIRI was associated with 30-day mortality in AFM and AKI, while only 24-h urine volume after admission and EF were identified as prognostic markers, indicating their potential value for early risk stratification and clinical decision-making in this high-risk population.

## Data Availability

The raw data supporting the conclusions of this article will be made available by the authors, without undue reservation.
