# Correction: Short-term outcome and prognostic factors in acute fulminant myocarditis with acute kidney injury patients

**DOI:** 10.3389/fmed.2026.1823823

**Published:** 2026-03-17

**Authors:** Frontiers Production Office

**Affiliations:** Frontiers Media SA, Lausanne, Switzerland

**Keywords:** acute fulminant myocarditis, AKI, prognostic factors, short-term outcome, SIRI

Affiliation “Department of Nephrology, Fuwai Central China Cardiovascular Hospital, Central China Fuwai Hospital of Zhengzhou University, Zhengzhou, Henan, China” was erroneously given as “Department of Nephrology, Fuwai Huazhong Cardiovascular Hospital, Central China Fuwai Hospital of Zhengzhou University, Zhengzhou, Henan, China.”

“Fuwai Huazhong Cardiovascular Hospital” was erroneously written instead of “Fuwai Central China Cardiovascular Hospital” in the article text.

A correction has been made to the abstract, paragraph 2:

“We retrospectively analyzed patients diagnosed with AFM and AKI at Fuwai Central China Cardiovascular Hospital between March 2018 and September 2024. Patients were divided into survival and death groups according to 30-day mortality.”

A correction has been made to the section **Materials and methods**, *Study design and participants*, Paragraph 5. The paragraph has been corrected to:

“Patients diagnosed with both AFM and AKI at the Fuwai Central China Cardiovascular Hospital (Cardiac Center of Henan Provincial People's Hospital) between March 2018 and September 2024 were eligible for inclusion. Patients were divided into a survival group and a death group based on 30-day mortality.”

The original version of this article has been updated.

